# An excellent deep-ultraviolet birefringent material based on [BO_2_]^∞^ infinite chains

**DOI:** 10.1038/s41377-022-00941-2

**Published:** 2022-08-12

**Authors:** Fangfang Zhang, Xinglong Chen, Min Zhang, Wenqi Jin, Shujuan Han, Zhihua Yang, Shilie Pan

**Affiliations:** 1 Research Center for Crystal Materials, CAS Key Laboratory of Functional Materials and Devices for Special Environments, Xinjiang Technical Institute of Physics and Chemistry, CAS, 40-1 South Beijing Road, Urumqi, 830011 China; 2 Center of Materials Science and Optoelectronics Engineering, University of Chinese Academy of Sciences, 100049 Beijing, China

**Keywords:** Photonic crystals, Photonic devices

## Abstract

Birefringent materials play indispensable roles in modulating the polarization of light and are vital in the laser science and technology. Currently, the design of birefringent materials operating in the deep-ultraviolet region (DUV, *λ* ≤200 nm) is still a great challenge. In this work, we developed a new DUV birefringent crystal LiBO_2_ based on [BO_2_]^∞^ infinite chains in the Li-B-O system, which simultaneously achieves the shortest UV cutoff edge (164 nm) and the largest birefringence (≥0.168 at 266 nm) among all the reported borate-based DUV birefringent materials. Single crystals of LiBO_2_ with dimensions up to Ø55 × 34 mm^3^ were grown by the Czochralski method, providing access to large-sized single crystal with low cost. Moreover, it has a high laser damage threshold and stable physicochemical properties. These outstanding characters unambiguously support that LiBO_2_ can be an excellent birefringent material for DUV application.

## Introduction

Birefringent crystals are important materials in modern laser and polarizing technology owing to their vital function to modulate the polarization of light^1–5^. Various birefringent crystals including YVO_4_^6^, TiO_2_^7^, LiNbO_3_^8^, CaCO_3_^9^, and *α*-BaB_2_O_4_ (*α*-BBO)^10^ are commercially available to fabricate optical devices operating over the wavelength regions from ultraviolet (UV) to mid-infrared (mid-IR). Nowadays, the deep-ultraviolet (DUV, *λ* ≤ 200 nm) laser technology has been greatly accelerated due to the significant advancements of nonlinear optical (NLO) crystals^11–18^ and laser frequency conversion techniques, that enable the output wavelengths below 200 nm^19–22^. Consequently, the demand for polarization devices based on DUV birefringent crystals is extremely urgent. In general, two prerequisites should preferably be combined for a DUV birefringent crystal. First, the UV cutoff edge (*λ*_cutoff_) should be as short as possible to achieve wide transparent range and high transmittance in the DUV region. A relatively high *λ*_cutoff_ would result in a narrow DUV transparent range (from *λ*_cutoff_ to 200 nm), besides, the transmittance in the spectral range that is close to *λ*_cutoff_ would be low, therefore, the working spectral range is limited^23,24^. Second, a sufficient birefringence is preferred. A small birefringence leads to a small beam splitting angle of the polarizing prism, which is not conducive to the compactness of the whole device^25^. In addition, from a viewpoint of application, other requirements should include high laser damage threshold (LDT), stable physicochemical properties, and suitable for large size crystal growth, etc.^26–28^. However, few commercial birefringent crystals can satisfy the requirements simultaneously, and the exploration of high performance DUV birefringent materials is still a great challenge.

Borate is one of the best material systems to explore DUV birefringent crystals^29–33^. Specifically, the strong covalent B−O bonds are ideal for the transmission of DUV light and the [BO_3_] units with sp² hybridization possess large polarizability anisotropy, of which the coplanar alignment is conducive to large birefringence^34^. Besides the commercial *α*-BBO crystal, some other borate birefringent crystals based on different anionic groups of [BO_3_]^35^, [B_2_O_5_]^36^, or [B_3_O_6_]^37–40^ were reported. In our previous work, we proposed that the [BO_2_]^∞^ infinite chain composed of corner-connected [BO_3_] units can serve as an optimal functional unit for DUV birefringent materials design. A [BO_2_]^∞^-based alkaline-earth metal borate, Ca(BO_2_)_2_, possessing both large birefringence and wide band gap was developed^41^. With the in-depth study of the [BO_2_]^∞^-based crystals, we realized that the Li-B-O system is even superior because of the following reasons: (i) Li has the largest electronegativity among alkali- and alkaline earth-metals, thus a shorter UV cutoff edge can be expected. (ii) lithium borate, such as the famous NLO crystal LiB_3_O_5_ (LBO)^42,43^, usually possesses desired high LDT that is important for practical applications. (iii) lithium borates have a low melting point, which can make it easy to grow crystal. (iv) the small ionic radius of Li is beneficial to increase the [BO_3_] density and thereby enhance the birefringence.

Guided by these ideas, we successfully developed a new [BO_2_]^∞^-based birefringent crystal LiBO_2_, which pushes the thresholds of *λ*_cutoff_ to 164 nm and a birefringence to larger than 0.168 at 266 nm, achieving the best among all the reported borate-based DUV birefringent crystals. The theoretical analyses reveal that good coplanarity of the [BO_2_]^∞^ chains (i.e., a small dihedral angle (DA) between adjacent [BO_3_] planes), as well as the large density of the [BO_3_] units leads to the large birefringence. Intriguingly, LiBO_2_ is a congruent melting compound with a relatively low melting point, and does not exhibit first-order phase transition, which make it easier for growing large crystal. Moreover, the crystal has a high LDT and good physicochemical stability as well as a low production cost. These excellent attributes suggest that LiBO_2_ is a superb birefringent material working in the DUV region.

## Results

**Fig. 1:**
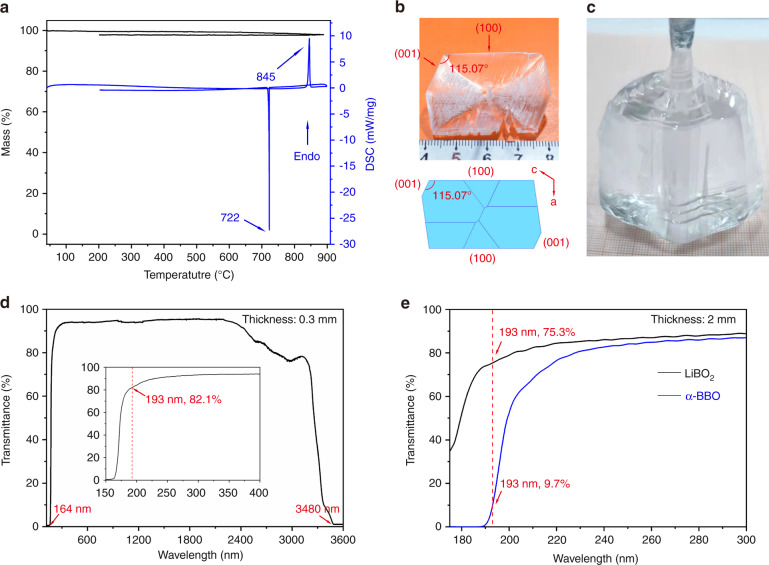
Thermal property, as-grown single crystals, and transmittance spectra of LiBO_2_. **a** TG-DSC curves of LiBO_2_. **b** As-grown LiBO_2_ crystal via top-seeded growth method from the stoichiometric melt (top) and the theoretical morphology viewed along *b*-axis (bottom). **c** As-grown LiBO_2_ crystal via the Czochralski method using [010]-oriented seed. **d** Transmission spectra of LiBO_2_. **e** Comparison of transmission spectra of LiBO_2_ and *α*-BBO

The thermal study of LiBO_2_ is shown in Fig. 1a. TG curve shows that there is scarcely any weight loss in the temperature up to 900 °C. Meanwhile, an endothermic peak at 845 °C and an exothermic peak at 722 °C are observed from the heating and cooling curves, respectively, which do not show any evidence of a first-order phase transition. The powder X-ray diffraction (XRD) pattern of the solidified melt agrees with that of the initial LiBO_2_ powder (Fig. S1). These results indicate the congruent melting feature of LiBO_2_, therefore, large single crystals, in principle, can be grown from stoichiometric melts. Figure 1b shows a LiBO_2_ crystal grown from the stoichiometric melt by the top-seeded growth method. The distinguishable crystal facets are (100) and (001), which are in accordance with the predicted growth morphology according to the Bravais−Friedel and Donnay−Harker (BFDH) theory^44^. Subsequently, colorless, high optical quality LiBO_2_ crystal with sizes up to Ø55 × 34 mm^3^ (Fig. 1c) was grown *via* the Czochralski method after a series of growth parameters optimizations within a short development period. It is worth to note that the crystal growth does not require a vacuum or inert atmosphere as the cases of MgF_2_ and YVO_4_ while the growth temperature is much lower than those of *α*-BBO and Ca(BO_2_)_2_^41,45–47^. The relatively low growth temperature can not only reduce thermal stress induced defects and shorten the growth cycle, but also avoid many severe growth conditions such as high-power furnace and expensive iridium crucible, etc. Moreover, since there is no phase transition during the growth process, it is easier to obtain a large single crystal than *α*-BBO which suffers a first-order phase transition during the cooling process. Accordingly, the growth of LiBO_2_ can be relatively efficient, cheaper, and easier.

The transmittance measurement of LiBO_2_ (0.3 mm-thick plate, without coating) shows a wide transparency region of 164–3480 nm, and the transmittance at 193 nm is higher than 80% (Fig. 1d), which indicates that the application of LiBO_2_ can cover a broad spectral region from DUV to near-IR. To further confirm the DUV transparent capacity, *α*-BBO was measured for comparison under the same condition (2 mm-thick plates, without coating). From Fig. 1e, we can clearly see that both crystals show high optical transmittance from 220 to 300 nm, in contrast, the transmittance of LiBO_2_ below 200 nm is significantly higher than that of *α*-BBO (e.g., 75.3 % vs. 9.7 % at 193 nm). We would highlight that its superior DUV transparent range (164–200 nm) can cover multiple coherent light wavelengths of great interest (e.g., 177, 193 nm) for various applications including high resolution photoelectron spectroscopy and photolithography.

**Fig. 2:**
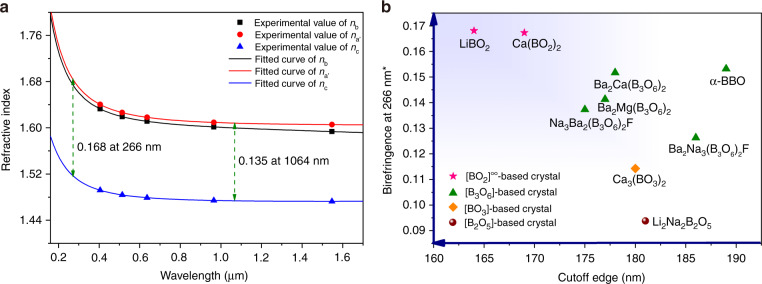
Refractive indices and birefringence of LiBO_2_. **a** Measured refractive indices and the fitted curves by the Sellmeier equations, where *n*_a′_ represents the refractive index perpendicular to the *b*-axis in the (001) plane, *n*_b_ and *n*_c_ are refractive index along *b*- and *c*-axes, respectively. **b** Comparison of borate DUV birefringent crystals based on different [BO] groups. *Data for LiBO_2_ are ∆*n*′ while for other crystals are calculated from Sellmeier equations (Refs. ^23,35–41^)

A (001) plate of LiBO_2_ was employed to evaluate the refractive indices and their dispersion via the prism coupling method. The refractive indices values at 405, 514, 636, 965, and 1547 nm are summarized in Table S1, where *n*_a′_ represents the refractive index perpendicular to the *b*-axis in the (001) plane. The included angle between *a*′ direction and the *a*-axis of the crystal is 25.07 ° according to the symmetry of LiBO_2_. Since LiBO_2_ crystallizes in the monoclinic system, only crystallographic *b*-axis coincides with one of the optical principal axes (*x*, *y*, *z*, corresponding to dielectric principal axes), while both *a-* and *c-*axes have a certain angle with other two optical principal axes of the crystal. Therefore, the real birefringence (∆*n* = *n*_z_ – *n*_x_) of the crystal should be larger than the current experimental values (∆*n*′ = *n*_a′_ – *n*_c_) because *n*_z_ should be close to but larger than *n*_a′_, while *n*_x_ should be close to but smaller than *n*_c_ in the case of LiBO_2_. Nonetheless, the experimental data were fitted to the following Sellmeier equations to preliminarily evaluate the birefringence of LiBO_2_ in a broader wavelength range,

$$n_{a^{\prime} }^2 = 2.567041 + \frac{{0.020843}}{{\lambda ^2 + 0.004167}} + 0.000750\lambda ^2$$

$$n_b^2 = 2.553004 + \frac{{0.019078}}{{\lambda ^2 + 0.002827}} - 0.009109\lambda ^2$$

$$n_c^2 = 2.159205 + \frac{{0.011337}}{{\lambda ^2 + 0.006320}} + 0.002135\lambda ^2$$

where *λ* is the wavelength expressed in micrometer. As shown in Fig. 2a, the difference between *n*_a′_ and *n*_c_ is pretty large, ∆*n*′ = *n*_a′_ – *n*_c_ = 0.168–0.135, in the wavelength range from 266 to 1064 nm. Figure 2b shows the comparison of LiBO_2_ with other borate-based DUV birefringent crystals^23,35–41^. The results indicate that LiBO_2_ possesses the shortest DUV cutoff edge and the largest birefringence simultaneously.

LDT is one of the most important parameters of a birefringent crystal for practical use. The LDT measurements (1064 nm, 10 ns, 10 Hz) of high optical quality LiBO_2_ and *α*-BBO crystals under the same conditions show that the LDT of LiBO_2_ is about 2.3 times higher than that of *α*-BBO. As the resistance ability to laser damage for the crystal is significantly dependent on the quality of the crystal, the higher LDT can be anticipated when the crystal quality is further improved in the future. A (001) plate of LiBO_2_ was exposed in the air at room temperature for one month to test the chemical stability. After that, the transparency and weight did not change, indicating that LiBO_2_ is stable in air and has good chemical stability. Mechanical hardness measurement on a (001) plate shows a Vickers hardness of 192 (HV0.3, 10 s), corresponding to a Mohs hardness of 3.3. This moderate hardness is beneficial for processing.

**Fig. 3:**
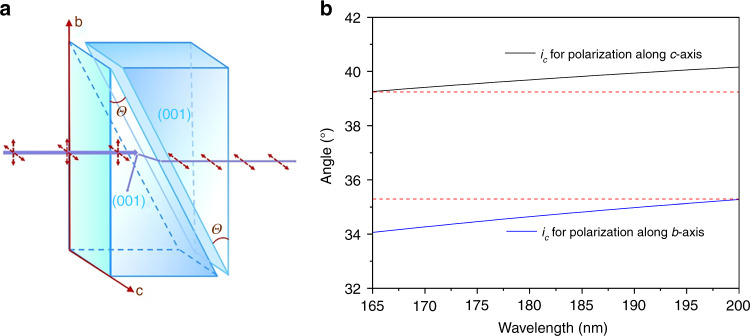
A design of optical polarization device based on LiBO_2_ crystal. **a** Schematic design of Glan-type polarizer based on LiBO_2_ crystal. **b** Critical angle of total internal reflection (*i*_c_) for polarization along *b*- and *c*-axes of LiBO_2_ in a wavelength ranging from 165 to 200 nm

The DUV transparent capacity along with the large birefringence of LiBO_2_ thus makes it an attractive candidate to design DUV Glan-type polarizer, which has been first realized in Ca(BO_2_)_2_ by our group^41^. Here we proposed a feasible design scheme for Glan polarizer based on LiBO_2_ crystal. As shown in Fig. 3a, the Glan polarizer is composed of two identical rectangular prisms. Considering the convenience for prism cutting and fabricating, the triangle base of the prism could be (001) face, which is a cleavage plane that is relatively easy to be obtained from the as-grown crystal. Besides, *bc*-plane that is perpendicular to (001) face can be selected as the face of normal incidence, in which the difference of refractive indices along two polarized directions (*b* and *c*), *n*_b_ – *n*_c_, is large enough in LiBO_2_ for Glan-type polarizer design. The hypotenuse lateral face of the left prism is for the selective transmission of the polarized light beams: the polarized light along *b*-axis would be totally reflected while only the polarized light along *c*-axis can transmit through the adjacent prism when the apex angle of the prism is appropriate. The critical angle for total internal reflection is determined by the following equation: *i*_c_ = arcsin(1/*n*_1_), where *n*_1_ is the refractive index along the polarized direction. To fulfill the requirement of DUV applications for LiBO_2_, we calculated the corresponding *i*_c_ for polarized light along *b-* and *c*-axes in the wavelength range of 165~200 nm, based on the refractive indices (*n*_b_ and *n*_c_) obtained from the fitted Sellmeier equations, and the results are plotted in Fig. 3b. In LiBO_2_ crystal, the apex angle (*θ*) of the prism should meet the condition as follows: 35.28° < *θ* < 39.26°, in the working spectral range of 165~200 nm. Note that the obtained tolerance range of apex angle is quite large (~4°) in a wide DUV range, which can bring great convenience in prism fabrication and practical application considering the case that light would be not perfectly normal to the incident plane. However, more experimental data of refractive indices at shorter wavelengths are needed in the future, which is currently limited by our instrument, to obtain a more accurate Sellmeier equations for the precise determination of the apex angle (*θ*) of the designed prism.

**Fig. 4:**
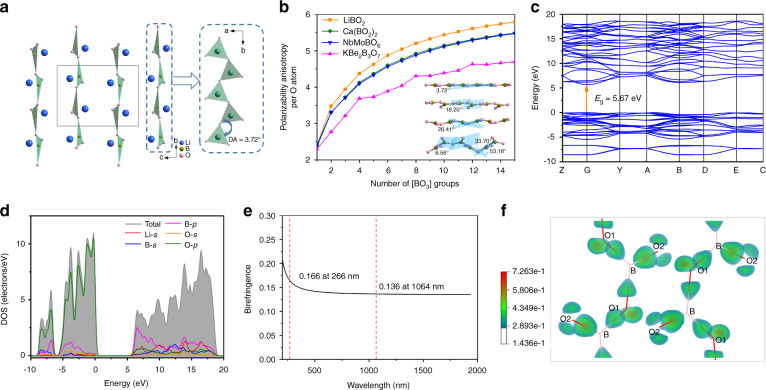
Crystal structure and theoretical calculations on structure-property relations of LiBO_2_. **a** Crystal structure of LiBO_2_ viewed along *a*-axis and the [BO_2_]^∞^ chains with a dihedral angle (DA) of 3.72 ° between adjacent [BO_3_] planes. **b** Average polarizability anisotropy per oxygen atom of [BO_2_]^∞^ chains with different dihedral angles. The number of [BO_3_] groups were chosen from 1 to 15 in the calculation. Structural models for Ca(BO_2_)_2_, NbMoBO_6_ and KBe_2_B_3_O_7_ are retrieved from Refs. ^41,56,57^, respectively. **c** Electron band structure of LiBO_2_. The arrow indicates the direct band gap. **d** Total and partial density of states (DOS) of LiBO_2_. **e** Calculated birefringence of LiBO_2_. **f** Electron density difference of LiBO_2_

Single crystal X-ray diffraction analysis verified that LiBO_2_ crystallizes in the monoclinic space group *P*2_1_/*c* (Table S2)^48^. In the structure, [BO_3_] triangles connect with each other by conner-sharing to form infinite [BO_2_]^∞^ chains arranging parallelly along the *b*-axis and the Li cations locate in the interstices. The dihedral angle (DA) between adjacent [BO_3_] planes in the [BO_2_]^∞^ chain is 3.72° (Fig. 4a), which is smaller than that of Ca(BO_2_)_2_ (18.2°), indicating an improved coplanarity of the [BO_2_]^∞^ chains in LiBO_2_. A theoretical calculation on polarizability anisotropy (*δ*) reveals that a better coplanarity of the [BO_2_]^∞^ chains results in a larger polarizability anisotropy, which is positively correlated to the birefringence (Fig. 4b). In addition, the density of [BO_3_] units of LiBO_2_ (26.86 nm^‒3^) is slightly larger than that of Ca(BO_2_)_2_ (25.97 nm^‒3^) owing to the shorter Li–O bonds, thus, LiBO_2_ possesses a larger birefringence (≥0.135 at 1064 nm) than that of Ca(BO_2_)_2_ (0.1225 at 1064 nm).

In order to further understand the relationship between the structure and optical properties, first principles calculations were carried out. LiBO_2_ has a direct band gap as illustriated in Fig. 4c. The total and partial densities of states (DOS) show that O-2p and B-2p states determine the valence band top and conduction band bottom (Fig. 4d), indicating that the band gap of LiBO_2_ is dominated by [BO_3_] units, that is, [BO_2_]^∞^ chains. The birefringence (∆*n*) of LiBO_2_ is calculated based on the relation between the wavelength and refractive index. Results show that LiBO_2_ is a negative biaxial crystal (*n*_z_ ‒ *n*_y_ < *n*_y_ ‒ *n*_x_), and the calculated birefringence is as high as 0.166 at 266 nm (Fig. 4e), which is comparable to the experimental value (∆*n*′ = 0.168 at 266 nm). Figure 4f illustrates the electron density difference of LiBO_2_ which clearly shows the electron distribution that can be attributed to the formation of B–O bonds. Response electron distribution anisotropy (REDA) analysis^49,50^ was also performed to examine the contribution of the anionic groups. The bonding electron density difference (Δ*ρ*) of the structural units, i.e., [BO_2_]^∞^ chains and [LiO_4_] tetrahedra, along the optical principal axes were calculated. From Table 1, we can see that the [BO_2_]^∞^ chains are the primary source of birefringence (~80%), which verify the effectiveness of our design strategy of DUV birefringent crystals using [BO_2_]^∞^ chains.

**Table 1:** Bonding electron difference (Δ*ρ*) of the bonds along the optical principal axes and contribution percent *w* (%) of the structural units that calculated by the response electron distribution anisotropy (REDA) model

Units	Δ*ρ*	*w* (%)
[LiO_4_]	0.0118	20.6
[BO_2_]^∞^	0.0454	79.4
Total	0.0572	/

## Discussion

In summary, we have successfully developed a new DUV birefringent crystal LiBO_2_ by screening the alkali- and alkaline earth-metal borates with the optimal [BO_2_]^∞^ infinite chains for achieving short DUV cutoff edge and large birefringence simultaneously. High-quality single crystals with maximum dimensions up to Ø55 × 34 mm^3^ have been obtained. Comprehensive experimental and theoretical studies show that LiBO_2_ exhibits both the largest birefringence (≥ 0.168 at 266 nm) and the shortest UV cutoff edge (164 nm) in comparison with other borate-based DUV birefringent materials. Moreover, it has a high LDT and stable physicochemical properties. Fascinatingly more, LiBO_2_ crystal is easier in crystal growth attributed to its multiple merits including congruent melting, free of first-order phase transition, rather low melting point (845 °C) among birefringent crystals, and achievable growth in an open system with ambient air. In addition, LiBO_2_ may have much lower production costs due to its low growth temperature, high production yields, and inexpensive raw materials. These outstanding characteristics clearly suggest that LiBO_2_ is an excellent DUV birefringent material. Future work will focus on the machining of prisms to evaluate the refractive index dispersion using the minimum deviation technique, as well as the design, manufacturing and assessment of polarizing devices.

## Materials and methods

### Synthesis, crystal growth and structure detection

Polycrystalline powder of LiBO_2_ was prepared by a conventional high temperature solid-state reaction method. A stoichiometric mixture of Li_2_CO_3_ and B_2_O_3_ was loaded into a platinum crucible, and then preheated at 700 °C for 24 h to decompose the carbonates. The product was ground thoroughly, and then gradually heated to 750 °C and held at this temperature for 72 h with several intermediate grindings and mixings. The phase purity of the sample was confirmed by powder XRD (Fig. S1).

Initially, top-seeded growth method was adopted to grow LiBO_2_ crystal for preliminary assessment of optical properties. A vertical-tube furnace equipped with heating element of resistance wire, pulling and rotating system and programable controller was employed. During the growths, the rotation speed was 5~10 rpm. After several crystal growth cycles, transparent crystals with well-developed facets were obtained. Oriented seed crystal was separated from the as-grown crystal for the subsequent Czochralski growth.

Large LiBO_2_ single crystals with higher optical qualities were grown by the Czochralski method using a furnace with RF heating. To compensate for the loss of B_2_O_3_ caused by the volatilization, 0.5 mol% excess of B_2_O_3_ was mixed with LiBO_2_ powder for melt preparation. Seed crystals with different orientations were tried and [010]-oriented seeds were finally adopted to optimize other growth parameters. Typically, the applied pulling rate was 0.2–1 mm·h^‒1^ and the rotation rate was 2–5 rpm. The diameters of the grown crystals were carefully controlled by power adjustment based on the observation during the crystal growth. After the crystals grew to a desired size, they were pulled up and separated from the melt manually. Afterwards, the crystals were cooled to room temperature at a rate of 5–30 °C·h^‒1^. The crystal growth was performed in ambient air condition and does not require a vacuum or inert atmosphere.

A single crystal of LiBO_2_ was selected for the structure determination by single-crystal XRD. The detailed method is presented in the Supplementary Information. Results show that LiBO_2_ crystallizes into the monoclinic space group of *P*2_1_/*c* with lattice parameters of *a* = 5.8529(8) Å, *b* = 4.3461(7) Å, *c* = 6.4630(9) Å, *β* = 115.071(10) °, *Z* = 4 (Table S2), in accordance with the data reported by Zachariasen^48^.

### Thermal analysis

The thermal gravimetric analysis (TG) analysis and differential scanning calorimetry (DSC) of LiBO_2_ were carried out on a simultaneous NETZSCH STA 449 F3 thermal analyzer instrument under a flowing N_2_ atmosphere. The crushed single crystal was enclosed in a platinum crucible, heated from 40 to 900 °C, and then cooled to 200 °C at a rate of 5 °C·min^−1^.

### Transmittance spectra

Transmittance spectra were measured on single crystals by vacuum UV analytical spectrophotometer under vacuum conditions (140~200 nm), SolidSpec-3700DUV spectrophotometer in a nitrogen gas atmosphere (175–2600 nm) and SHIMADZU IRAffinity-1 Fourier transform infrared spectrometer in the air (2500–25000 nm).

### Refractive indices determination

A (001) plate of LiBO_2_ was used to measure the refractive indices at the wavelengths of 405, 514, 636, 965, and 1547 nm, on the Metricon model 2010/M prism coupler (Metricon Co.) The accuracy of the measurements is estimated to be 2 × 10^−4^. The refractive indices along *a*′- and *b*-axes direction were measured by using transverse electric mode which tests the refractive indices parallel to the crystal plane. The refractive indices along *c*-axis were tested using transverse magnetic mode which tests the refractive indices perpendicular to the crystal plane.

### Laser damage threshold

The laser damage threshold of LiBO_2_ single crystal was measured on a pulsed Q-switched Nd:YAG laser (1064 nm, 10 ns, 10 Hz). The incident plane is (001) face that was optically polished. A commercial *α*-BBO crystal was measured under the same conditions for comparison. An optical convex lens was used to obtain the laser beam with a diameter of 1 mm. The damage was confirmed afterward by observing the irradiated sites under a microscope.

### Mechanical hardness

The Vickers hardness of a LiBO_2_ crystal with (001) face was measured using a DHV-1000 microhardness meter, with HV0.3 and a dwell time of 10 s. Five points were tested, and the average value was calculated as the final value. Mohs hardness (HM) was calculated from Vickers hardness (HV) by using the following equation: HM = 0.675(HV)1/3.

### Numerical calculation

The electronic structure and optical properties were calculated by employing CASTEP package^51^ based on density functional theory (DFT) with the norm-conserving pseudopotentials (NCPs)^52,53^. The exchange-correlation functionals were Perdew-Burke-Ernzerhof (PBE) functional within the generalized gradient approximation (GGA)^54^. The plane-wave energy cutoff was set at 750.0 eV. The *k*-point grid was generated as 4 × 4 × 3 using the Monkhorst-Pack grid parameters. The empty bands were set as 3 times of valence bands for the calculation of the optical properties. Because GGA method usually underestimates the bandgap, the scissors operators were utilized to shift the conduction bands to agree with the experimental band gap values, and then the refractive indexes were obtained by the real part of the dielectric function on the base of the Kramers-Kronig transform. The polarizability anisotropy of anionic groups was calculated using DFT implemented by the Gaussian09 package^55^. B3LYP (Becke, three-parameter, Lee-Yang-Parr) exchange-correlation functional with the Lee-Yang-Parr correlation functional at the 6-31G basis set in Gaussian was employed.

Birefringence is sensitive to the anisotropy of the response electron distribution, corresponding to REDA index $\zeta = \mathop {\sum }\nolimits_g \left[ {N_cZ_a\Delta \rho ^b/\left( {n_1E_o} \right)} \right]_g$ of the anionic groups contained in the same crystal^49^, where *N*_*c*_ is the coordination number of the nearest neighbor cations to the anion, *E*_*o*_ is the optical bandgap, $\Delta \rho ^b = \rho _{max}^b - \rho _{min}^b$, $\rho _{{{{\mathrm{max}}}}}^b$ and $\rho _{{{{\mathrm{min}}}}}^b$ are the maximum and minimum of the covalent electron density of the covalent bond on the optical principal axes of a crystal, and *n*_1_ is the minimum refractive index.

## Supplementary information

Supplementary Information for An Excellent Deep-Ultraviolet Birefringent Material Based on [BO2]∞ Infinite Chains
