# Supplementary material for: An excellent deep-ultraviolet birefringent material based on [BO2]∞ infinite chains
Source: Light Sci Appl. 2022 Aug 12;11:252. doi: 10.1038/s41377-022-00941-2 (PMC9372186; doi:10.1038/s41377-022-00941-2)
Supplement: Supplementary file 1 — Supplementary Information for An Excellent Deep-Ultraviolet Birefringent Material Based on [BO2]∞ Infinite Chains [file 41377_2022_941_MOESM1_ESM.docx]

*Supplementary Information for*

**An Excellent Deep-Ultraviolet Birefringent Material Based on** **[BO_2_]^∞^ Infinite Chains**

Fangfang Zhang^1,2,#^ , Xinglong Chen^1,2,#^, Min Zhang^1,2^, Wenqi Jin^1,2^, Shujuan Han^1,2^, Zhihua Yang^1,2^, and Shilie Pan^1,2,*^

^1^Research Center for Crystal Materials, CAS Key Laboratory of Functional Materials and Devices for Special Environments, Xinjiang Technical Institute of Physics and Chemistry, CAS, 40-1 South Beijing Road, Urumqi 830011, China

^2^Center of Materials Science and Optoelectronics Engineering, University of Chinese Academy of Sciences, Beijing 100049, China

^#^These authors contribute equally to this work

^*^To whom correspondence should be addressed. E-mail: slpan@ms.xjb.ac.cn

**Methodology**

Powder X-ray diffraction (XRD) measurement was performed at room temperature in the 2*θ* range of 10 - 70 ° with a scan step width of 0.02 ° and a fixed counting time of 1 s per step using a Bruker D2 ADVANNCE X-ray diffractometer equipped with a diffracted beam monochromator set for Cu Kα radiation (*λ* = 1.5418 Å).

Single-crystal XRD measurement was performed on an APEX II CCD diffractometer using graphite-monochromatic Mo *Kα* radiation (*λ* = 0.71073 Å) at 296(2) K and integrated with the SAINT program.^1^ Numerical absorption corrections were performed by using the SCALE program for area detector. All calculations were performed with programs from the SHELXTL package.^2^ All atoms were refined using full-matrix least-squares techniques, final least-squares refinement is on *F*_o_*^2^* with data having *F*_o_^2^ ≥ 2*σ* (*F*_o_^2^). The structure was checked with PLATON and no higher symmetries were found.^3^

**Table S1.** Experimental refractive indices of LiBO_2_ along three directions.

| Wavelength  (nm) | *n*_b_ | *n*_a’_ | *n*_c_ | △*n’*  (*n*_a’_ ‒ *n*_c_) |
| --- | --- | --- | --- | --- |
| 405 | 1.63274 | 1.64045 | 1.49202 | 0.14072 |
| 514 | 1.61927 | 1.62632 | 1.48381 | 0.13546 |
| 636 | 1.61126 | 1.61813 | 1.47908 | 0.13218 |
| 965 | 1.60155 | 1.60936 | 1.47421 | 0.12734 |
| 1547 | 1.59348 | 1.60547 | 1.47277 | 0.12071 |

**Table S2.** Crystal data and structure refinement for LiBO_2_.

| Empirical formula | LiBO_2_ |
| --- | --- |
| Formula weight | 49.75 g mol^−1^ |
| Temperature | 296(2) K |
| Crystal system | Monoclinic |
| Space group, *Z* | *P*2_1_/*c*, 4 |
| Unit cell dimensions | *a* = 5.8529(8) Å |
|  | *b* = 4.3461(7) Å |
|  | *c* = 6.4630(9) Å  *β* = 115.071(10)° |
| Volume | 148.91(4) Å^3^ |
| Density (calculated) | 2.219 g cm^‒3^ |
| *F*(000) | 96 |
| Crystal size | 0.203 mm × 0.119 mm × 0.117 mm |
| Theta range for data collection | 3.84 to 27.48 ° |
| Limiting indices | ‒ 7 ≤ h ≤ 7, ‒ 5 ≤ k ≤ 5, ‒ 8 ≤ l ≤ 8 |
| Reflections collected / unique | 1295 / 342 [*R*(int) = 0.0168] |
| Completeness to theta = 27.48 ° | 100.0 % |
| Refinement method | Full-matrix least-squares on *F*^2^ |
| Goodness-of-fit on *F*^2^ | 1.110 |
| Final *R* indices [*F*_o_^2^ > 2*σ*(*F*_o_^2^)]^[a]^ | *R*_1_ = 0.0265, *wR*_2_ = 0.0731 |
| *R* indices (all data)^[a]^ | *R*_1_ = 0.0300, *wR*_2_ = 0.0755 |
| Extinction coefficient | 0.00(2) |
| Largest diff. peak and hole | 0.177 and ‒ 0.193 e Å^‒3^ |

^[a]^*R*_1_ = Σ||*F*_o_| ‒ |*F*_c_||/Σ|*F*_o_| and *wR*_2_ = [Σ*w*(*F*_o_^2^ – *F*_c_^2^)^2^ / Σ*wF*_o_^4^]^1/2^ for *F*_o_^2^ > 2*σ*(*F*_o_^2^)

**Fig. S1** Experimental and simulated powder X-ray diffraction (XRD) patterns of LiBO_2_.

**
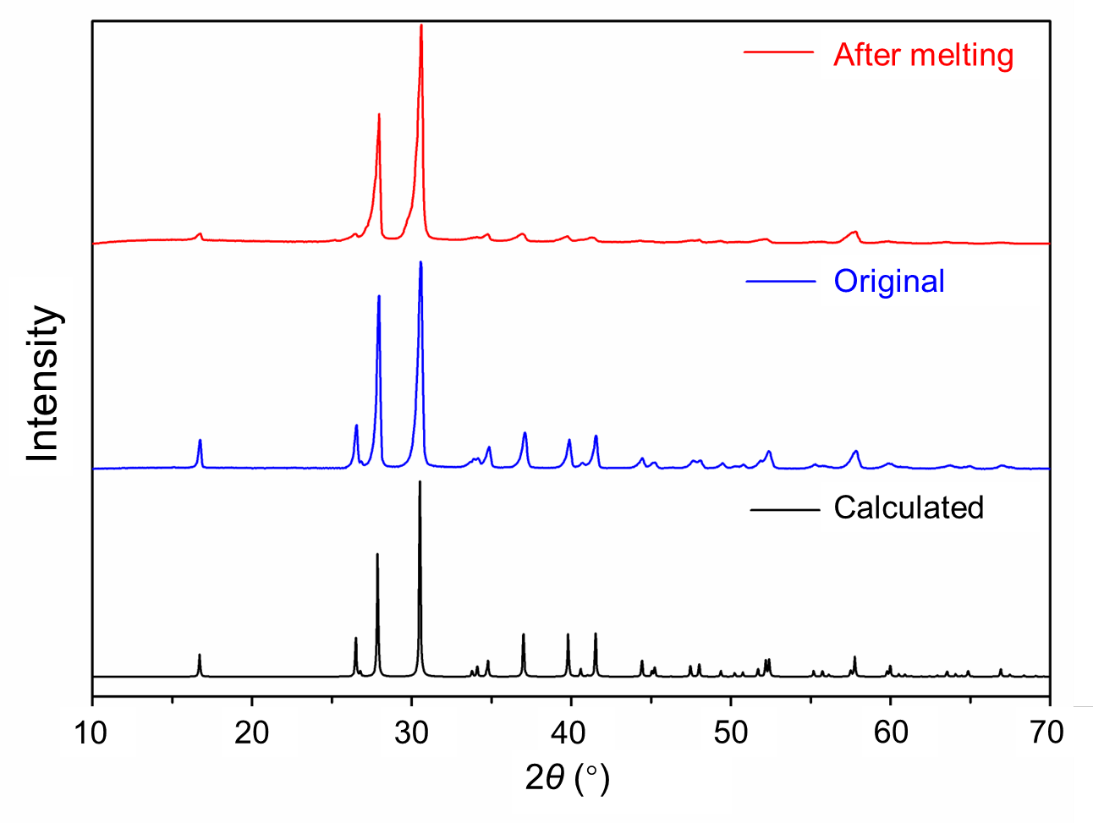
**

**References**

1. SAINT, version 7.60A; Bruker Analytical X-ray Instruments, Inc., Madison, WI, 2008.

2. Sheldrick, G. M. SHELXTL, version 6.14; Bruker Analytical Xray Instruments, Inc., Madison, WI, 2003.

3. Spek, A. L. Single-crystal structure validation with the program PLATON. *J. Appl. Crystallogr.* **36**, 7−13 (2003).
